# Complete genome sequence of *Paraburkholderia* sp. strain DD10, a hyphospheric bacterium isolated from *Rhizophagus irregularis* mycelium

**DOI:** 10.1128/mra.00286-25

**Published:** 2026-02-13

**Authors:** Jennifer Simm, Alexandra Haouy, Mohamed Zouaoui, Hugo Guerrero, Thomas Rey, Christophe Roux

**Affiliations:** 1 University of Toulouse, CNRS, Toulouse INP, LRSV 27091 , Toulouse, France; 2 DE SANGOSSE 339690 , Pont-du-Casse, France; California State University San Marcos, San Marcos, California, USA

**Keywords:** *Paraburkholderia*, hyphosphere, arbuscular mycorrhizal fungi

## Abstract

This announcement describes the genome of *Paraburkholderia* sp. strain DD10, a bacterium originating from meadow soil that associates with hyphae of the arbuscular mycorrhizal fungus *Rhizophagus irregularis*. Oxford nanopore sequencing and genome assembly yielded a 7,684,915 bp sequence containing 6,941 predicted genes, comprising one chromosome, one chromid, and one plasmid.

## ANNOUNCEMENT

The hyphosphere, defined as the bacterial microbiota associated with mycelium, constitutes a highly specialized environment where bacterial diversity is strongly influenced by the fungus (1). *Paraburkholderia* strain DD10, a gram-negative, rod-shaped, motile bacterium, was isolated from the hyphosphere of the extraradical mycelium of the arbuscular mycorrhizal (AM) fungus—Glomeromycotina*—Rhizophagus irregularis*. This strain is a valuable tool to investigate the underlying mechanisms involved in bacteria-AM-fungus interaction.

This bacterium was isolated using a trapping assay. Flax plants (*Linum usitatissimum*), Eurodor variety, were inoculated with 1,000 spores from a liquid inoculum of *R. irregularis* DAOM197198 during sowing. The culture was established in a double-compartment pot system, where only the fungal hyphae could access the second compartment through a 10 µm mesh. A fine and coarse zeolite mix was added as a substrate in both compartments. Plants were watered with 0.5× Long Ashton solution (2), while the second compartment was enriched with phytate (0.3 mmol) and nitrate (2.5 mmol). A leachate obtained from a meadow soil (42°31′49.3″ N, 2°59′06.3″ E) was introduced into the fungal compartment. After 8 weeks, bacteria thriving on the fungal hyphae were isolated using a protocol adapted from Zhang et al. (3). The hypospheric bacterial suspension was washed with and serially diluted in 10% tryptic soy broth to obtain single bacterial colonies per plate. Bacteria were tested for their abilities to dephosphorylate phytate through a simple test on an agar and phytate plate (4) for 10 days at 24°C and to grow along the hyphae of *R. irregularis* in a dark room for 2–3 weeks at 24°C. The growth was assessed in a double-compartment system *in vitro*, where an OD 0.1 dilution of a bacterial culture was inoculated at the start of the second compartment. DD10 displays both properties and could use the hyphae to access phosphorus patches throughout heterogeneous soils (5). DD10 DNA was isolated from a Luria-Bertani liquid culture incubated for 72 h at 28°C with agitation. The cell suspension was centrifuged, and the pellet was treated with the NucleoBond kit from Macherey-Nagel with the Set III and the AGX100 columns to obtain purified DNA. The sequencing library was prepared using the Rapid Barcoding Kit 96 V14 (Oxford Nanopore Technologies) following the manufacturer’s protocol. No shearing or size selection was performed. Sequencing was performed on an Oxford Nanopore MinION using an R10.4 flow cell. Data acquisition was carried out with MinKNOW. Base calling and read quality control, including error correction and adapter trimming, were performed using Dorado 0.6.1 on a computing cluster (Bioinfo Genotoul, Toulouse Occitanie). The sequencing yielded a read *N*_50_ of 7.7 kb and 136,564 raw reads. The *de novo* assembly was made using the long-read assembler Flye 2.9.2 (6). The genome was not rotated. Annotation was performed using the NCBI Prokaryotic Genome Annotation Pipeline (PGAP) 6.10 (7).

Three contigs were produced, corresponding to a 4 Mbp circular DNA strand, a 3.4 Mbp chromid (8), and a 212 kbp plasmid. The resulting features of the genome are presented in Table 1. The closest taxon was revealed to be *Paraburkholderia terricola* (Taxonomy ID 169427: SAMN05192548) with 97%, and the identification was carried out using TYGS (9). A total of 6,941 genes were predicted, and Operon mapper v.1 was used to identify the number of operons (10). The completeness of the genome was analyzed with BUSCO 5.8.3 (11) against the 688 genes found in the *Burkholderiales* group, showing a 100% completeness with two genes present in double copies. Default parameters were used except where otherwise noted for all software.

**TABLE 1:** Genomic information on *Paraburkholderia* sp. DD10

Feature	Value for *Paraburkholderia* DD10
Total length (bp)	7,684,915
% GC content	63.5%
Predicted genes	6,941
Predicted protein-coding genes	6,710
Operon sRNA	4,070

## Data Availability

The raw sequencing reads, complete genome assembly, and annotation are available at NCBI under the following accession numbers: SRP575236, SAMN47339132, and GCF_049177125.1.
